# Scrub Typhus Reemergence in the Maldives

**DOI:** 10.3201/eid0912.030212

**Published:** 2003-12

**Authors:** Michael D. Lewis, Abdul Azeez Yousuf, Kriangkrai Lerdthusnee, Ahmed Razee, Kirkvitch Chandranoi, James W. Jones

**Affiliations:** * Armed Forces Research Institute of Medical Sciences, Bangkok, Thailand; † Ministry of Health, Malé, Republic of Maldives; ‡ Indira Gandhi Memorial Hospital, Malé, Republic of Maldives

**Keywords:** Scrub typhus [C01.252.400.790.790.850], *Orientia tsutsugamushi* [B03.440.650.650.650.550], rickettsia infections [C01.252.400.790.790.790], Maldives (Indian ocean islands [Z01.600]), tropical medicine [G02.403.919]

## Abstract

In summer 2002, an outbreak of febrile illness began in the Maldives in the Indian Ocean. Through April 2003, officials recorded 168 cases with 10 deaths. The Armed Forces Research Institute of Medicine in Bangkok confirmed *Orientia tsutsugamushi* and conducted a joint investigation with the Ministry of Health, Maldives. These cases of scrub typhus were the first in the Maldives since World War II.

In the summer of 2002, an outbreak of febrile illness began in the Republic of Maldives, a nation of 26 coral atolls straddling the equator in the Indian Ocean. The Ministry of Health, Republic of Maldives, intensified surveillance efforts, and scrub typhus was clinically suspected by the beginning of September. From May 28, 2002, through April 17, 2003, the Ministry of Health has recorded 168 suspected and confirmed cases with 10 deaths. The disease appears to have a focus (74 cases) in the Gaafu Dhaalu Atoll, just north of the equator, including 57 cases and three deaths on Gadhdhoo Island (year 2000 population = 1,719) (Table 1, Figure).

**Table 1:** Total cases, laboratory-confirmed cases, and deaths in the Maldives by atoll^a^ and island (listed from north to south) from May 28, 2002, to April 27, 2003^b^

Atoll	Island	Total cases	Laboratory-confirmed cases^b^	Deaths
Haa Alifu	Thakandhoo	1	0	0
	Filladhoo	1	1	1
	Naivaadhoo	1	0	0
	Baarah	1	0	0
		**4**	**1**	**1**
Haa Dhaalu	Hanimaadhoo	1	0	0
	Nolhivaranfaru	1	0	0
	Kurinbi	1	0	0
	Vaikaradhoo	2	0	1
		**5**	**0**	**1**
Shaviyani	Kanditheem	2	1	1
	Noomaraa	1	0	0
	Maroshi	1	0	0
		**4**	**1**	**1**
Raa	Alifushi	1	0	0
	Rasgetheem	2	0	1
	Inguraidhoo	10	0	1
	Kinolhas	1	0	0
		**14**	**0**	**2**
Baa	Thulhaadhoo	**2**	**0**	**0**
Kaafu	Malé	**4**	**0**	**0**
North Alifu	Rasdhoo	3	0	0
	Ukulhas	2	2	0
	Feridhoo	4	1	1
		**9**	**3**	**1**
South Alifu	Himendhoo	**1**	**0**	**0**
Dhaalu	Rinbudhoo	2	1	0
	Gemendhoo	1	1	0
		**3**	**2**	**0**
Thaa	Vilufushi	1	0	0
	Dhiyamigili	1	0	0
	Guraidhoo	2	0	0
		**4**	**0**	**0**
Laamu	Isdhoo	1	0	0
	Gan	1	0	0
	Kadhdhoo	2	0	0
		**4**	**0**	**0**
Gaafu Alifu	Viningili	4	0	0
	Koodhoo	1	0	0
	Dhaandhoo	6	1	0
	Maamendhoo	1	0	0
	Nilandhoo	3	0	0
	Gemanafushi	4	1	1
	Kanduhulhudhoo	2	0	0
		**21**	**2**	**1**
Gaafu Dhaalu	Thinadhoo	4	3	0
	Maathadaa	4	0	0
	Gadhdhoo	57	12	3
	Vaadhoo	2	1	0
	Fiyoaree	4	0	0
	Fares	5	0	0
		**76**	**16**	**3**
Gnaviyani	Fuvahmulah	**9**	**0**	**0**
Seenu (Addu)	Hithadhoo (Gan)	5	0	0
	Hulhudhoo	1	0	0
	Feydhoo	2	0	0
		**8**	**0**	**0**
				
Total		**168**	**38**	**10**

^a^Numbers in bold indicate atoll totals. ^b^Confirmed either at the Armed Forces Research Institute of Medicine (see Table 2) or by enzyme-linked immunosorbent assay at Indira Gandhi Memorial Hospital in Malé.

**Figure:**
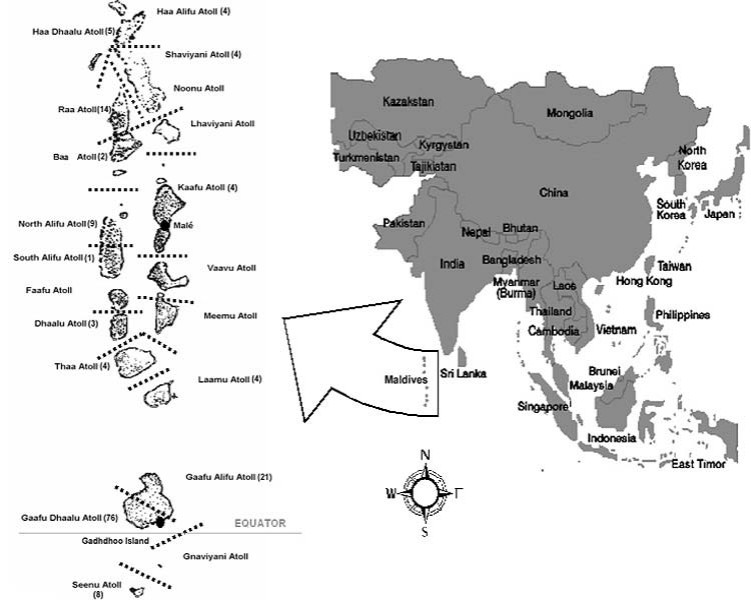
Republic of Maldives by atoll (total number of cases, May 28, 2002, to April 27, 2003, in parentheses next to atoll name).

The last cases of scrub typhus in the Maldives were recorded by British troops during World War II (*1*). Following their arrival in October 1941 on Gan Island, Addu Atoll, the Royal Marines suffered an outbreak of 42 cases. In 1942, the British had another 582 cases, 382 in 1943, 92 in 1944, and none in 1945. J.R. Audy, a British Army physician during the war, reported that rats were numerous in the area and *Trombicula* (now *Leptotrombidium*) *deliense* were found in a variety of habitats (*1*). Scrub typhus was also documented by an Indian Army survey after the war (*2*). According to S.L. Kalra, General Headquarters–India raised a field typhus team that joined the Scrub Typhus Research Laboratory under the South East Asia Command in 1945. After the war, the team continued investigations of rickettsial diseases, surveying 23 locations from Addu Atoll, Maldives, to the Himalayas. A published report noted that beech rain forests in the Addu Atoll possessed both the host and vector of scrub typhus and the rickettsia, whereas the atoll was negative for Q fever and murine, epidemic, and tick typhus (*2*).

Scrub typhus is a chigger-borne disease caused by the rickettsia *Orientia tsutsugamushi* (*3*–*6*). The disease is endemic in Asia and remains an important public health problem (*1*,*3*–*6*). Scrub typhus was the most notable rickettsiosis affecting U.S. troops and had a higher mortality rate than any other infectious disease in WWII in the China-Burma-India theatre of operations (*5*). Larval trombiculid mites become infected with *O. tsutsugamushi* during feeds on their usual hosts, small rodents. Rodents influence mite population density and serve as a reservoir for the agent, although transovarial vertical transmission of the agent in mites is the dominant maintenance mechanism. Humans become infected when they accidentally encroach in an area where the chigger-rodent cycle is occurring, most often areas of low-lying scrub brush or transitional vegetation. *O. tsutsugamushi* is transmitted to humans when a chigger attaches itself to the skin in the search for food (*3*–*7*).

Scrub typhus often appears as a nonspecific febrile illness. Diagnosis and surveillance can be challenging, particularly in the absence of advanced laboratory diagnostic techniques (*3*–*6*). Typical symptoms include fever, headache, rash, and lymphadenopathy (*3*–*7*). The presence of an eschar is pathognomonic, but it is typically overlooked or misdiagnosed (*3*,*4*,*6*). Pulmonary involvement frequently occurs in mild cases and is the principal cause of death in severe disease (*3*,*4*). Before antimicrobial drugs, case-fatality rates ranged from 5% to 40% (*3*–*7*).

Since the departure of the British, no cases of scrub typhus have been recorded in the Maldives. Following the deaths of three adolescents on Gadhdhoo Island in July–August 2002, the Director General of Health Services, Ministry of Health, contacted researchers at the Armed Forces Research Institute of Medicine (AFRIMS) in Bangkok, Thailand, for assistance with diagnostics and disease control. In September 2002, AFRIMS received specimens from 31 patients: 28 serologic and 4 whole blood samples (both serum and whole blood received from one patient). Serologic testing was done by indirect fluorescent antibody (IFA), indirect immunoperoxidase (IIP), and enzyme-linked immunosorbent assay (ELISA) testing. The presence of the *O. tsutsugamushi* DNA was tested for by polymerase chain reaction (PCR) in the whole blood samples received.

Procedures for the IIP assay were those described by Suto (*8*) and Land et al. (*9*). The IFA was modified from the microimmunofluorescence methods described by Bozeman and Elisberg (*10*) and Robinson et al. (*11*). PanBio scrub typhus immunoglobulin (Ig) M and IgG rapid immunochromatographic assays were obtained (PanBio, Brisbane, Australia) and used according to the insert (Insert-2-SCT-25S, 13 July 2000). AFRIMS identified 14 of 28 serologic samples as positive for *O. tsustugamushi* infection (Table 2).

**Table 2:** Testing results performed at the Armed Forces Research Institute of Medical Sciences

	IIP^a^	IFA^b^		ELISA^c^
IgM (+)	14	15		14
IgG (+)	24	24		24
IgM (+) + IgG (+)	14	15		14
Total samples tested	28	28		28
PCR^e^ and agarose gel electrophoresis positive results:			2 of 4	

^a^Indirect immunoperoxidase test (positive if titer was ≥1:400) (*15*). ^b^ Indirect immunofluorescent antibody test (positive if titer was ≥1:50) (*15*). ^c^Enzyme-linked immunosorbent assay (positive if PanBio units >11) (*15*). ^d^Positive immunoglobulin (Ig) M plus IgG results indicate active *Orientia tsustugamushi* infection. IgG without IgM indicates the persistence of IgG after a primary infection but with no active infection. The absence of both IgG and IgM indicates no exposure or a very early stage of infection (*15*). ^e^Polymerase chain reaction for *O. tsustugamushi* DNA (*13*).

PCR is considered the most sensitive and specific method available for detecting the rickettsial DNA of *O. tsustugamushi* (*12*,*13*). Primary PCR and nested PCR were used for DNA amplification of *O. tsutsugamushi* (*13*). AFRIMS identified *O. tsutsugamushi* DNA in two of the four whole blood samples provided.

At the invitation of the Minister of Health, Republic of Maldives, AFRIMS researchers conducted a joint consultation with health officials in the South Huvadhu Atoll and Malé, the capital. Assessments were conducted as well as meetings with the communities, community leaders, and healthcare workers involved in controlling the disease. Clinical, diagnostic, and entomologic advice was presented to healthcare providers at the Indira Gandhi Memorial Hospital in Malé in addition to providing the hospital laboratory with training and supplies to conduct ELISAs.

An onsite evaluation showed that while Gadhdhoo is mostly residential, rodent habitats exist. Wood for cooking fuel is commonly stored in backyards near kitchens, which are often outdoors. An island clean-up campaign to consolidate trash sites into two established locations appears to have been successfully implemented by the island leaders, but some yards still are prime rodent habitats, along with several common areas of low-lying transitional grassy areas. Many residents travel by boat several hundred meters to the uninhabited Gan Island to cultivate food such as yams. Gan Island is a textbook example of a chigger habitat. Trails leading to cultivated areas are bordered by tall grass and brushy habitat. Islanders reported that Gan Island was inhabited many years ago, but a mysterious fatal illness fell over the island and caused the residents to flee to Gadhdhoo decades ago.

Because health officials and the island leadership have been proactive in controlling the scrub typhus situation through rodent control and vegetation clean-up on Gadhdhoo Island, only two rodents were trapped (*14*) by local officials, both which were identified by AFRIMS as *Rattus remotus*. One rat was found to have 15 chiggers in the ear that were identified as *L. deliense* but were found not to be harboring *O. tsutsugamushi* when tested by PCR.

Three questions remain concerning the current outbreak of scrub typhus in the Maldives: 1) where did the scrub typhus come from; 2) why is scrub typhus now occurring; and 3) why is there a disproportionate focus on Gadhdhoo? Because of the transovarian and transtadial transmission of *O. tsutsugamushi* in mites (*3*–*7*), this pathogen has likely always been in the Maldives, but cases have been unrecognized since the British wartime occupation because of a lack of surveillance and diagnostics infrastructure.

The second and third questions are closely related. Scrub typhus, at least in Japan, can appear in cycles and completely disappear in between (*6*). Scrub typhus in the Maldives was known to occur in the 1940s, quite possibly due to the impact of introducing naïve British soldiers into a wartime occupational situation with a large intrusion on the natural habitat of rodents and chiggers (*1*). Not until the summer of 2002, when three adolescent deaths occurred on Gadhdhoo Island, was confirmatory laboratory diagnosis sought. The Ministry of Health then began educating healthcare providers and making the public aware of the situation.

What was different on Gadhdhoo Island that caused its population to suffer a disproportionate number of cases? In addition to the occurrence of a possible scrub typhus cycle, a large die-off of wild and domestic cats occurred in 2000. Cats are the small island’s only natural predator of rodents. A subsequent large increase in the rodent population occurred over the next 2 years, leading to an increased interaction between the human and rodent populations. Additionally, in February 2002, the island leaders began an aggressive campaign to clean up trash sites and yards. Increased exposures to rodent populations as a result of disturbances of rodent habitats are well known to result in increases in the incidence of scrub typhus (*1*,*3*–*6*).

Through a combined effort between the Ministry of Health and AFRIMS, we have documented the reappearance of scrub typhus in the Maldives 58 years after the last reported cases. While the disease has possibly existed in the Maldives for many years, it is now being clinically recognized and diagnosed through the efforts of the Ministry of Health.
